# The iron phosphate NaBaFe_2_(PO_4_)_3_

**DOI:** 10.1107/S1600536808023040

**Published:** 2008-07-31

**Authors:** Mourad Hidouri, Hasna Jerbi, Mongi Ben Amara

**Affiliations:** a Faculté des Sciences de Monastir, 5019 Monastir, Tunisia

## Abstract

A new iron phosphate, sodium barium diiron tris­(phosphate), NaBaFe_2_(PO_4_)_3_, has been synthesized by the flux method and shown to exhibit the well known langbeinite type structure. The Na, Ba and Fe atoms all lie on threefold axes, while the P and O atoms occupy general positions, one of the O atoms being disordered over two positions, with site occupancy factors of *ca* 0.7 and 0.3. The [Fe_2_(PO_4_)_3_]_∞_ framework consists of FeO_6_ octa­hedra sharing all their corners with the PO_4_ tetra­hedra. The Na^+^ and Ba^2+^ cations are almost equally distributed over two distinct cavities, in which they occupy slightly different positions.

## Related literature

For related literature, see: Baur (1974 ▶); Moffat (1978 ▶); Padhi *et al.* (1997 ▶); Shannon (1976 ▶). For the structure of langbeinite, see Zemann & Zemann (1957 ▶); Battle *et al.* (1986 ▶, 1988 ▶).

## Experimental

### 

#### Crystal data

NaBaFe_2_(PO_4_)_3_
*M* *_r_* = 556.94
Cubic, 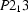

*a* = 9.796 (1) Å
*V* = 940.1 (3) Å^3^
*Z* = 4
Mo *K*α radiation
μ = 7.82 mm^−1^
*T* = 293 (2) K
0.1 × 0.1 × 0.1 mm

#### Data collection

Enraf–Nonius CAD4 diffractometer
Absorption correction: ψ scan (North *et al.*, 1968 ▶) *T*_min_ = 0.35, *T*_max_ = 0.46
2114 measured reflections
657 independent reflections
644 reflections with *I* > 2σ(*I*)
*R*_int_ = 0.082
2 standard reflections frequency: 120 min intensity decay: 1%

#### Refinement

*R*[*F* ^2^ > 2σ(*F* ^2^)] = 0.025
*wR*(*F* ^2^) = 0.059
*S* = 0.92
657 reflections
70 parameters
4 restraints
Δρ_max_ = 0.57 e Å^−3^
Δρ_min_ = −0.49 e Å^−3^
Absolute structure: Flack (1983 ▶), 123 Friedel pairs
Flack parameter: −0.03 (3)

### 

Data collection: *CAD-4 EXPRESS* (Enraf–Nonius, 1994 ▶); cell refinement: *CAD-4 EXPRESS*; data reduction: *XCAD4* (Harms & Wocadlo, 1995 ▶); program(s) used to solve structure: *SHELXS97* (Sheldrick, 2008 ▶); program(s) used to refine structure: *SHELXL97* (Sheldrick, 2008 ▶); molecular graphics: *DIAMOND* (Brandenburg, 1998 ▶); software used to prepare material for publication: *SHELXL97*.

## Supplementary Material

Crystal structure: contains datablocks I, global. DOI: 10.1107/S1600536808023040/br2076sup1.cif

Structure factors: contains datablocks I. DOI: 10.1107/S1600536808023040/br2076Isup2.hkl

Additional supplementary materials:  crystallographic information; 3D view; checkCIF report

## Figures and Tables

**Table 1:** Selected bond angles (°)

O4*B*^i^—Fe2—O1^ii^	89.8 (8)
O3—P—O4*A*	115.1 (3)

Symmetry codes: (i) 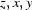; (ii) 
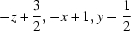
.

**Fig. 1.**
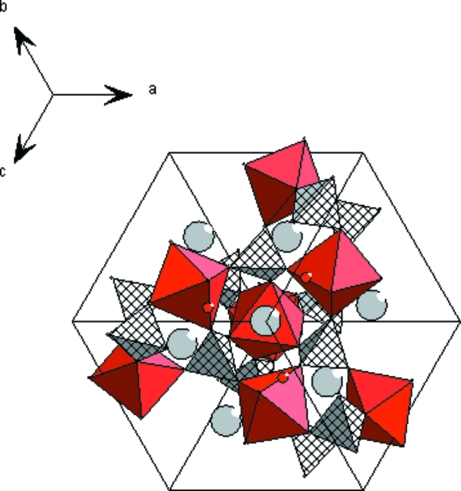
A projection of the structure along the [111] direction.

**Fig. 2.**
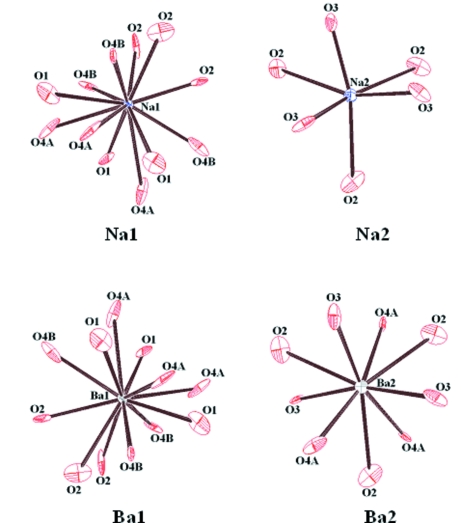
The environments of the Na and Ba sites showing the anisotropic atomic displacements at the 50% level.

## supplementary crystallographic information

### Comment

Iron phosphates are of increasing interst because of their potential applications in various fields ranging from catalysis (Moffat, 1978) to ionic conductivity (Padhi *et al.*, 1997). Moreover, these materials are very attractive in terms of basic reasearch because they exhibit a rich structural chemistry owing to the possible (+2/+3) mixed valence of iron and its tendency to exhibit various coordination polyhedra.

The title cmpound, sodium barium diiron phosphate NaBaFe_2_(PO_4_)_3_ was isolated during a systematic investigation of the Na_2_O–*M*O–Fe_2_O_3_–P_2_O_5_ systems where *M* is a divalent cation. Its structure (Fig. 1) exhibits a three-dimensional [Fe_2_(PO_4_)_3_]_∞_ framework built up from corner-sharing FeO_6_ octahedra and PO_4_ tetrahedra. Each octahedron is linked to six adjacent tetrahedra and reciprocally each tetrahedron is connected to four neighboring octahedra. This framework delimits two sorts of large cavities, statistically occupied by the Na^+^ and Ba^2+^ cations.

The two symmetry distinct FeO_6_ octahedra contained in this structure are somewhat distorted as indicated by the Fe—O distances ranging from 1.963 (5) to 1.991 (4) Å. The average <Fe—O> distances of 1.986 Å for Fe(1) and 1.973 Å for Fe(2) are slightly lower than the value 2.03 Å predicted by Shannon for octahedral Fe^3+^ ions (Shannon, 1976).

The PO_4_ tetrahedron is strongly distorted with P—O distances scattering from 1.47 (2) to 1.547 (7) Å. Corresponding average value of 1.511 Å agrees with those frequently observed in anhydrous monophosphates (Baur, 1974).

The Na^+^ and Ba^2+^ cations are statistically distributed over two distinct cavities in which they occupy slightly different positions and have partial occupancies of 0.47, 0.53, 0.53 and 0.47 for Na(1), Ba(1), Na(2) and Ba(2), respectively. The environments of these cations (Fig. 2) were determined assuming all cation-oxygen distances are shorter than the shortest to next cationic site. Each of the Na(1), Ba(1) and Ba(2) environments consists of nine O atoms with cation-oxygen distances in the ranges 2.76 (2)–3.04 (2) Å, 2.753 (7)–2.950 (6) Å and 2.722 (5)–3.047 (7) Å for Na(1), Ba(1) and Ba(2), respectively. The Na(2) environment consists of six O atoms with Na—O distances varying from 2.604 (8) and 3.004 (6) Å.

The as-described structure is closely related to the langbeinite-like phosphates KBa*M*_2_(PO_4_)_3_ (*M* = Fe, Cr) (Battle *et al.*, 1986, 1988). However, it differs by the fact that the atom O4, which occupies a single site in the potassium phosphates, is, in the title compound, statistically occupying two distinct positions, O4A and O4B which exhibit partial occupancies of 0.7 and 0.3, respectively. These different values can be explained by the fact that the O4A site is occupied if it is bonded to Na(1), Ba(1) or Ba(2) whereas the O4B site is occupied if it is bonded to Na(1) or Ba(1).

### Experimental

Single crystals of the title compund were grown in a flux of sodium dimolybdate Na_2_Mo_2_O_7_ with an atomic ratio P:Mo = 6:1. A starting mixture of 1.071 g of Na_2_CO_3_, 1.993 g of BaCO_3_, 8.162 g of Fe(NO_3_)_3_.9H_2_O, 4.002 g of (NH_4_)_2_HPO_4_ and 1.454 g of MoO_3_ was dissolved in nitric acid and the obtained solution was evaporated to dryness. The dry residue was transferred into a platinum crucible and then heated up 600°C to decompose H_2_O and NH_3_. In a second step, the sample was melted for 1 h at 900°C and then cooled down to room temperature with a 10° h^-1^ rate. The final product, obtained after washing with warm water to dissolve the flux is essentially composed of pink and prismatic shaped crystals. Their qualitative elemental analysis using electron microprobe analysis indicated the presence of Na, Ba, Fe and P and no impurity elements have been detected.

### Refinement

The Ba and Fe atoms were located by direct methods and the remaining atoms were found by successive difference Fourier maps. All atomic positions were refined anisotropically.

### Crystal data

**Table:**

NaBaFe_2_(PO_4_)_3_	*Z* = 4
*M**_r_* = 556.94	*F*_000_ = 1040
Cubic, *P*2_1_3	*D*_x_ = 3.935 Mg m^−^^3^
Hall symbol: P 2ac 2ab 3	Mo *K*α radiation λ = 0.71073 Å
*a* = 9.796 (1) Å	Cell parameters from 25 reflections
*b* = 9.796 (1) Å	θ = 9.0–13.0º
*c* = 9.796 (1) Å	µ = 7.82 mm^−^^1^
α = 90º	*T* = 293 (2) K
β = 90º	Prism, pink
γ = 90º	0.1 × 0.1 × 0.1 mm
*V* = 940.1 (3) Å^3^	

### Data collection

**Table:**

Enraf–Nonius CAD-4 diffractometer	*R*_int_ = 0.082
Radiation source: fine-focus sealed tube	θ_max_ = 29.9º
Monochromator: graphite	θ_min_ = 2.9º
*T* = 293(2) K	*h* = −1→13
ω/2θ scans	*k* = −1→13
Absorption correction: ψ scan(North *et al.*, 1968)	*l* = −1→13
*T*_min_ = 0.35, *T*_max_ = 0.46	2 standard reflections
2114 measured reflections	every 120 min
657 independent reflections	intensity decay: 1%
644 reflections with *I* > 2σ(*I*)	

### Refinement

**Table:**

Refinement on *F*^2^	Secondary atom site location: difference Fourier map
Least-squares matrix: full	*w* = 1/[σ^2^(*F*_o_^2^) + 5.7579*P*] where *P* = (*F*_o_^2^ + 2*F*_c_^2^)/3
*R*[*F*^2^ > 2σ(*F*^2^)] = 0.025	(Δ/σ)_max_ = 0.002
*wR*(*F*^2^) = 0.059	Δρ_max_ = 0.57 e Å^−^^3^
*S* = 0.92	Δρ_min_ = −0.49 e Å^−^^3^
657 reflections	Extinction correction: SHELXL97 (Sheldrick, 2008), Fc^*^=kFc[1+0.001xFc^2^λ^3^/sin(2θ)]^-1/4^
70 parameters	Extinction coefficient: 0.0145 (15)
4 restraints	Absolute structure: Flack (1983), with how many Friedel pairs?
Primary atom site location: structure-invariant direct methods	Flack parameter: −0.03 (3)

### Special details

**Table:**

Geometry. All e.s.d.'s (except the e.s.d. in the dihedral angle between two l.s. planes) are estimated using the full covariance matrix. The cell e.s.d.'s are taken into account individually in the estimation of e.s.d.'s in distances, angles and torsion angles; correlations between e.s.d.'s in cell parameters are only used when they are defined by crystal symmetry. An approximate (isotropic) treatment of cell e.s.d.'s is used for estimating e.s.d.'s involving l.s. planes.

### Fractional atomic coordinates and isotropic or equivalent isotropic displacement parameters (Å^2^)

**Table:**

	*x*	*y*	*z*	*U*_iso_*/*U*_eq_	Occ. (<1)
Na1	0.9427 (12)	0.9427 (12)	0.9427 (12)	0.0144 (3)	0.4738 (16)
Ba1	0.92953 (9)	0.92953 (9)	0.92953 (9)	0.0144 (3)	0.5262 (16)
Na2	0.6862 (8)	0.6862 (8)	0.6862 (8)	0.0232 (4)	0.5262 (16)
Ba2	0.70555 (8)	0.70555 (8)	0.70555 (8)	0.0232 (4)	0.4738 (16)
Fe1	0.35313 (6)	0.85313 (6)	0.64687 (6)	0.0104 (2)	
Fe2	0.91362 (6)	0.08638 (6)	0.58638 (6)	0.0101 (2)	
P	0.03742 (10)	0.77099 (11)	0.62578 (10)	0.0068 (2)	
O1	0.9926 (5)	0.9134 (4)	0.6562 (7)	0.0461 (14)	
O2	0.9463 (5)	0.6999 (6)	0.5243 (5)	0.0440 (13)	
O3	0.1846 (4)	0.7653 (6)	0.5752 (5)	0.0368 (12)	
O4A	0.0112 (7)	0.6985 (10)	0.7629 (8)	0.0389 (18)	0.701 (4)
O4B	0.0527 (17)	0.672 (2)	0.738 (2)	0.0389 (18)	0.299 (4)

### Atomic displacement parameters (Å^2^)

**Table:**

	*U*^11^	*U*^22^	*U*^33^	*U*^12^	*U*^13^	*U*^23^
Na1	0.0144 (3)	0.0144 (3)	0.0144 (3)	−0.0019 (3)	−0.0019 (3)	−0.0019 (3)
Ba1	0.0144 (3)	0.0144 (3)	0.0144 (3)	−0.0019 (3)	−0.0019 (3)	−0.0019 (3)
Na2	0.0232 (4)	0.0232 (4)	0.0232 (4)	0.0016 (3)	0.0016 (3)	0.0016 (3)
Ba2	0.0232 (4)	0.0232 (4)	0.0232 (4)	0.0016 (3)	0.0016 (3)	0.0016 (3)
Fe1	0.0104 (2)	0.0104 (2)	0.0104 (2)	0.0001 (2)	−0.0001 (2)	−0.0001 (2)
Fe2	0.0101 (2)	0.0101 (2)	0.0101 (2)	0.0024 (2)	0.0024 (2)	−0.0024 (2)
P	0.0066 (4)	0.0073 (4)	0.0064 (4)	−0.0005 (3)	−0.0028 (3)	0.0010 (3)
O1	0.045 (3)	0.0170 (19)	0.076 (4)	0.0191 (19)	−0.015 (3)	−0.015 (2)
O2	0.029 (2)	0.061 (3)	0.042 (3)	0.003 (2)	−0.025 (2)	−0.031 (2)
O3	0.0120 (16)	0.063 (3)	0.035 (2)	−0.0091 (18)	0.0085 (16)	−0.023 (2)
O4A	0.020 (4)	0.066 (5)	0.031 (3)	0.020 (3)	0.015 (3)	0.040 (3)
O4B	0.020 (4)	0.066 (5)	0.031 (3)	0.020 (3)	0.015 (3)	0.040 (3)

### Geometric parameters (Å, °)

**Table:**

Na1—O1^i^	2.864 (12)	Ba2—O3^xiii^	2.772 (5)
Na1—O1	2.864 (12)	Ba2—O3^xiv^	2.772 (5)
Na1—O1^ii^	2.864 (12)	Ba2—O2^ii^	2.952 (5)
Na1—O4B^iii^	2.86 (3)	Ba2—O2	2.952 (5)
Na1—O4B^iv^	2.86 (3)	Ba2—O2^i^	2.952 (5)
Na1—O4B^v^	2.86 (3)	Ba2—O4A^vi^	3.047 (7)
Na1—O4A^vi^	3.045 (17)	Ba2—O4A^vii^	3.047 (7)
Na1—O4A^vii^	3.045 (17)	Ba2—O4A^viii^	3.047 (7)
Na1—O4A^viii^	3.045 (17)	Fe1—O2^ii^	1.979 (4)
Na1—O2^ix^	2.763 (17)	Fe1—O2^xv^	1.979 (4)
Na1—O2^x^	2.763 (17)	Fe1—O2^xvi^	1.979 (4)
Na1—O2^xi^	2.763 (17)	Fe1—O3^xiii^	1.990 (4)
Ba1—O1^i^	2.753 (7)	Fe1—O3	1.990 (4)
Ba1—O1	2.753 (7)	Fe1—O3^xvii^	1.990 (4)
Ba1—O1^ii^	2.753 (7)	Fe1—Ba1^xviii^	3.6878 (19)
Ba1—O4B^iii^	2.89 (3)	Fe1—Ba2^iv^	3.7867 (6)
Ba1—O4B^iv^	2.89 (3)	Fe1—Ba2^xv^	3.7867 (6)
Ba1—O4B^v^	2.89 (3)	Fe2—O4B^xix^	1.946 (18)
Ba1—O4A^vi^	2.902 (10)	Fe2—O4B^ii^	1.946 (18)
Ba1—O4A^vii^	2.902 (10)	Fe2—O4B^xii^	1.946 (18)
Ba1—O4A^viii^	2.902 (10)	Fe2—O1^xx^	1.984 (5)
Ba1—O2^ix^	2.950 (6)	Fe2—O1^xxi^	1.984 (5)
Ba1—O2^x^	2.950 (6)	Fe2—O1^xxii^	1.984 (5)
Ba1—O2^xi^	2.950 (6)	Fe2—O4A^xix^	1.982 (7)
Na2—O3^xii^	2.604 (8)	Fe2—O4A^ii^	1.982 (7)
Na2—O3^xiii^	2.604 (8)	Fe2—O4A^xii^	1.982 (7)
Na2—O3^xiv^	2.604 (8)	P—O4B	1.470 (18)
Na2—O2	3.004 (6)	P—O1^xxiii^	1.493 (4)
Na2—O2^i^	3.004 (6)	P—O2^xxiii^	1.506 (4)
Na2—O2^ii^	3.004 (6)	P—O3	1.526 (4)
Ba2—O3^xii^	2.772 (5)	P—O4A	1.541 (7)
			
O1^i^—Na1—O1	94.9 (5)	O2^ix^—Ba1—O2^xi^	55.17 (13)
O1^i^—Na1—O1^ii^	94.9 (5)	O2^x^—Ba1—O2^xi^	55.17 (13)
O1—Na1—O1^ii^	94.9 (5)	O3^xii^—Na2—O3^xiii^	100.1 (3)
O1^i^—Na1—O4B^iii^	58.0 (4)	O3^xii^—Na2—O3^xiv^	100.1 (3)
O1—Na1—O4B^iii^	79.6 (4)	O3^xiii^—Na2—O3^xiv^	100.1 (3)
O1^ii^—Na1—O4B^iii^	151.3 (7)	O3^xii^—Na2—O2	83.46 (16)
O1^i^—Na1—O4B^iv^	151.3 (7)	O3^xiii^—Na2—O2	158.5 (4)
O1—Na1—O4B^iv^	58.0 (4)	O3^xiv^—Na2—O2	58.53 (12)
O1^ii^—Na1—O4B^iv^	79.6 (4)	O3^xii^—Na2—O2^i^	58.53 (12)
O4B^iii^—Na1—O4B^iv^	118.89 (19)	O3^xiii^—Na2—O2^i^	83.46 (16)
O1^i^—Na1—O4B^v^	79.6 (4)	O3^xiv^—Na2—O2^i^	158.5 (4)
O1—Na1—O4B^v^	151.3 (7)	O2—Na2—O2^i^	115.68 (18)
O1^ii^—Na1—O4B^v^	58.0 (4)	O3^xii^—Na2—O2^ii^	158.5 (4)
O4B^iii^—Na1—O4B^v^	118.89 (19)	O3^xiii^—Na2—O2^ii^	58.53 (12)
O4B^iv^—Na1—O4B^v^	118.89 (19)	O3^xiv^—Na2—O2^ii^	83.46 (16)
O1^i^—Na1—O4A^vi^	46.9 (2)	O2—Na2—O2^ii^	115.68 (18)
O1—Na1—O4A^vi^	107.0 (6)	O2^i^—Na2—O2^ii^	115.68 (18)
O1^ii^—Na1—O4A^vi^	49.5 (3)	O3^xii^—Ba2—O3^xiii^	92.09 (15)
O1^i^—Na1—O4A^vii^	49.5 (3)	O3^xii^—Ba2—O3^xiv^	92.09 (15)
O1—Na1—O4A^vii^	46.9 (2)	O3^xiii^—Ba2—O3^xiv^	92.09 (15)
O1^ii^—Na1—O4A^vii^	107.0 (6)	O3^xii^—Ba2—O2^ii^	148.54 (14)
O1^i^—Na1—O4A^viii^	107.0 (6)	O3^xiii^—Ba2—O2^ii^	57.63 (12)
O1—Na1—O4A^viii^	49.5 (3)	O3^xiv^—Ba2—O2^ii^	81.64 (14)
O1^ii^—Na1—O4A^viii^	46.9 (2)	O3^xii^—Ba2—O2	81.64 (14)
O4A^vi^—Na1—O4A^viii^	81.1 (5)	O3^xiii^—Ba2—O2	148.54 (14)
O4A^vii^—Na1—O4A^viii^	81.1 (5)	O3^xiv^—Ba2—O2	57.63 (12)
O1^i^—Na1—O2^ix^	97.97 (19)	O2^ii^—Ba2—O2	118.95 (3)
O1—Na1—O2^ix^	104.0 (2)	O3^xii^—Ba2—O2^i^	57.63 (12)
O1^ii^—Na1—O2^ix^	156.0 (6)	O3^xiii^—Ba2—O2^i^	81.64 (14)
O4B^iv^—Na1—O2^ix^	97.9 (6)	O3^xiv^—Ba2—O2^i^	148.54 (14)
O4B^v^—Na1—O2^ix^	104.6 (6)	O2^ii^—Ba2—O2^i^	118.95 (3)
O4A^vi^—Na1—O2^ix^	134.2 (2)	O2—Ba2—O2^i^	118.95 (3)
O4A^vii^—Na1—O2^ix^	96.81 (16)	O3^xii^—Ba2—O4A^vi^	104.95 (16)
O4A^viii^—Na1—O2^ix^	144.2 (3)	O3^xiii^—Ba2—O4A^vi^	83.7 (2)
O1^i^—Na1—O2^x^	156.0 (6)	O3^xiv^—Ba2—O4A^vi^	162.54 (16)
O1—Na1—O2^x^	97.97 (19)	O2^ii^—Ba2—O4A^vi^	81.80 (17)
O1^ii^—Na1—O2^x^	104.0 (2)	O2—Ba2—O4A^vi^	127.8 (2)
O4B^iii^—Na1—O2^x^	104.6 (6)	O2^i^—Ba2—O4A^vi^	47.59 (16)
O4B^iv^—Na1—O2^x^	49.4 (4)	O3^xii^—Ba2—O4A^vii^	83.7 (2)
O4B^v^—Na1—O2^x^	97.9 (6)	O3^xiii^—Ba2—O4A^vii^	162.54 (16)
O4A^vi^—Na1—O2^x^	144.2 (3)	O3^xiv^—Ba2—O4A^vii^	104.95 (16)
O4A^vii^—Na1—O2^x^	134.2 (2)	O2^ii^—Ba2—O4A^vii^	127.8 (2)
O4A^viii^—Na1—O2^x^	96.81 (16)	O2—Ba2—O4A^vii^	47.59 (16)
O2^ix^—Na1—O2^x^	59.2 (4)	O2^i^—Ba2—O4A^vii^	81.80 (17)
O1^i^—Na1—O2^xi^	104.0 (2)	O4A^vi^—Ba2—O4A^vii^	81.1 (3)
O1—Na1—O2^xi^	156.0 (6)	O3^xii^—Ba2—O4A^viii^	162.54 (17)
O1^ii^—Na1—O2^xi^	97.97 (19)	O3^xiii^—Ba2—O4A^viii^	104.95 (16)
O4B^iii^—Na1—O2^xi^	97.9 (6)	O3^xiv^—Ba2—O4A^viii^	83.7 (2)
O4B^iv^—Na1—O2^xi^	104.6 (6)	O2^ii^—Ba2—O4A^viii^	47.59 (16)
O4A^vi^—Na1—O2^xi^	96.81 (16)	O2—Ba2—O4A^viii^	81.80 (17)
O4A^vii^—Na1—O2^xi^	144.2 (3)	O2^i^—Ba2—O4A^viii^	127.8 (2)
O4A^viii^—Na1—O2^xi^	134.2 (2)	O4A^vi^—Ba2—O4A^viii^	81.1 (3)
O2^ix^—Na1—O2^xi^	59.2 (4)	O4A^vii^—Ba2—O4A^viii^	81.1 (3)
O2^x^—Na1—O2^xi^	59.2 (4)	O2^ii^—Fe1—O2^xv^	87.3 (2)
O1^i^—Ba1—O1	100.10 (14)	O2^ii^—Fe1—O2^xvi^	87.3 (2)
O1^i^—Ba1—O1^ii^	100.10 (14)	O2^xv^—Fe1—O2^xvi^	87.3 (2)
O1—Ba1—O1^ii^	100.10 (14)	O2^ii^—Fe1—O3^xiii^	88.26 (18)
O1^i^—Ba1—O4B^iii^	58.8 (4)	O2^xv^—Fe1—O3^xiii^	89.2 (2)
O1—Ba1—O4B^iii^	80.9 (4)	O2^xvi^—Fe1—O3^xiii^	174.5 (2)
O1^ii^—Ba1—O4B^iii^	158.5 (4)	O2^ii^—Fe1—O3	174.5 (2)
O1^i^—Ba1—O4B^iv^	158.5 (4)	O2^xv^—Fe1—O3	88.26 (18)
O1—Ba1—O4B^iv^	58.8 (4)	O2^xvi^—Fe1—O3	89.2 (2)
O1^ii^—Ba1—O4B^iv^	80.9 (4)	O3^xiii^—Fe1—O3	95.0 (2)
O4B^iii^—Ba1—O4B^iv^	116.8 (2)	O2^ii^—Fe1—O3^xvii^	89.2 (2)
O1^i^—Ba1—O4B^v^	80.9 (4)	O2^xv^—Fe1—O3^xvii^	174.5 (2)
O1—Ba1—O4B^v^	158.5 (4)	O2^xvi^—Fe1—O3^xvii^	88.26 (18)
O1^ii^—Ba1—O4B^v^	58.8 (4)	O3^xiii^—Fe1—O3^xvii^	95.0 (2)
O4B^iii^—Ba1—O4B^v^	116.8 (2)	O3—Fe1—O3^xvii^	95.0 (2)
O4B^iv^—Ba1—O4B^v^	116.8 (2)	O4B^xix^—Fe2—O4B^ii^	80.8 (9)
O1^i^—Ba1—O4A^vi^	49.19 (16)	O4B^xix^—Fe2—O4B^xii^	80.8 (9)
O1—Ba1—O4A^vi^	114.25 (19)	O4B^ii^—Fe2—O4B^xii^	80.8 (9)
O1^ii^—Ba1—O4A^vi^	51.94 (17)	O4B^xix^—Fe2—O1^xx^	169.5 (6)
O1^i^—Ba1—O4A^vii^	51.94 (17)	O4B^ii^—Fe2—O1^xx^	89.8 (8)
O1—Ba1—O4A^vii^	49.19 (16)	O4B^xii^—Fe2—O1^xx^	93.0 (5)
O1^ii^—Ba1—O4A^vii^	114.25 (19)	O4B^xix^—Fe2—O1^xxi^	93.0 (5)
O1^i^—Ba1—O4A^viii^	114.25 (19)	O4B^ii^—Fe2—O1^xxi^	169.5 (6)
O1—Ba1—O4A^viii^	51.94 (17)	O4B^xii^—Fe2—O1^xxi^	89.8 (8)
O1^ii^—Ba1—O4A^viii^	49.19 (16)	O1^xx^—Fe2—O1^xxi^	95.5 (3)
O4A^vi^—Ba1—O4A^viii^	86.1 (2)	O4B^xix^—Fe2—O1^xxii^	89.8 (8)
O4A^vii^—Ba1—O4A^viii^	86.1 (2)	O4B^ii^—Fe2—O1^xxii^	93.0 (5)
O1^i^—Ba1—O2^ix^	96.20 (14)	O4B^xii^—Fe2—O1^xxii^	169.5 (6)
O1—Ba1—O2^ix^	102.04 (15)	O1^xx^—Fe2—O1^xxii^	95.5 (3)
O1^ii^—Ba1—O2^ix^	149.64 (14)	O1^xxi^—Fe2—O1^xxii^	95.5 (3)
O4B^iii^—Ba1—O2^ix^	47.5 (4)	O1^xx^—Fe2—O4A^xix^	168.6 (3)
O4B^iv^—Ba1—O2^ix^	93.1 (4)	O1^xxi^—Fe2—O4A^xix^	77.4 (3)
O4B^v^—Ba1—O2^ix^	99.2 (4)	O1^xxii^—Fe2—O4A^xix^	94.1 (3)
O4A^vi^—Ba1—O2^ix^	132.27 (18)	O4B^xix^—Fe2—O4A^ii^	78.2 (6)
O4A^vii^—Ba1—O2^ix^	95.97 (17)	O4B^ii^—Fe2—O4A^ii^	15.9 (5)
O4A^viii^—Ba1—O2^ix^	141.66 (18)	O4B^xii^—Fe2—O4A^ii^	95.9 (8)
O1^i^—Ba1—O2^x^	149.64 (14)	O1^xx^—Fe2—O4A^ii^	94.1 (3)
O1—Ba1—O2^x^	96.20 (14)	O1^xxi^—Fe2—O4A^ii^	168.6 (3)
O1^ii^—Ba1—O2^x^	102.04 (15)	O1^xxii^—Fe2—O4A^ii^	77.4 (3)
O4B^iii^—Ba1—O2^x^	99.2 (4)	O4A^xix^—Fe2—O4A^ii^	94.0 (3)
O4B^iv^—Ba1—O2^x^	47.5 (4)	O1^xx^—Fe2—O4A^xii^	77.4 (3)
O4B^v^—Ba1—O2^x^	93.1 (4)	O1^xxi^—Fe2—O4A^xii^	94.1 (3)
O4A^vi^—Ba1—O2^x^	141.66 (18)	O1^xxii^—Fe2—O4A^xii^	168.6 (3)
O4A^vii^—Ba1—O2^x^	132.27 (18)	O4A^xix^—Fe2—O4A^xii^	94.0 (3)
O4A^viii^—Ba1—O2^x^	95.97 (17)	O4A^ii^—Fe2—O4A^xii^	94.0 (3)
O2^ix^—Ba1—O2^x^	55.17 (13)	O4B—P—O1^xxiii^	119.9 (10)
O1^i^—Ba1—O2^xi^	102.04 (15)	O4B—P—O2^xxiii^	104.3 (11)
O1—Ba1—O2^xi^	149.64 (14)	O1^xxiii^—P—O2^xxiii^	112.9 (3)
O1^ii^—Ba1—O2^xi^	96.20 (14)	O4B—P—O3	97.0 (6)
O4B^iii^—Ba1—O2^xi^	93.1 (4)	O1^xxiii^—P—O3	112.2 (3)
O4B^iv^—Ba1—O2^xi^	99.2 (4)	O2^xxiii^—P—O3	109.2 (3)
O4B^v^—Ba1—O2^xi^	47.5 (4)	O4B—P—O4A	20.6 (6)
O4A^vi^—Ba1—O2^xi^	95.97 (16)	O1^xxiii^—P—O4A	102.0 (4)
O4A^vii^—Ba1—O2^xi^	141.66 (18)	O2^xxiii^—P—O4A	105.3 (4)
O4A^viii^—Ba1—O2^xi^	132.27 (18)	O3—P—O4A	115.1 (3)

Symmetry codes: (i) *y*, *z*, *x*; (ii) *z*, *x*, *y*; (iii) *y*+1/2, −*z*+3/2, −*x*+1; (iv) −*x*+1, *y*+1/2, −*z*+3/2; (v) −*z*+3/2, −*x*+1, *y*+1/2; (vi) *y*, *z*, *x*+1; (vii) *x*+1, *y*, *z*; (viii) *z*, *x*+1, *y*; (ix) *y*+1/2, −*z*+3/2, −*x*+2; (x) −*x*+2, *y*+1/2, −*z*+3/2; (xi) −*z*+3/2, −*x*+2, *y*+1/2; (xii) −*y*+3/2, −*z*+1, *x*+1/2; (xiii) −*z*+1, *x*+1/2, −*y*+3/2; (xiv) *x*+1/2, −*y*+3/2, −*z*+1; (xv) *x*−1/2, −*y*+3/2, −*z*+1; (xvi) −*y*+1, *z*+1/2, −*x*+3/2; (xvii) *y*−1/2, −*z*+3/2, −*x*+1; (xviii) −*x*+3/2, −*y*+2, *z*−1/2; (xix) −*x*+1, *y*−1/2, −*z*+3/2; (xx) −*z*+3/2, −*x*+1, *y*−1/2; (xxi) −*y*+2, *z*−1/2, −*x*+3/2; (xxii) *x*, *y*−1, *z*; (xxiii) *x*−1, *y*, *z*.
